# Supplementary material for: Artificial Weathering Mechanisms of Uncoated Structural Polyethylene Terephthalate Fabrics with Focus on Tensile Strength Degradation
Source: Materials (Basel). 2021 Jan 29;14(3):618. doi: 10.3390/ma14030618 (PMC7866265; doi:10.3390/ma14030618)
Supplement: Supplementary file 1 [file materials-14-00618-s001.pdf]

materials

Supplementary material

Artificial Weathering Mechanisms of Uncoated Structural Polyethylene Terephthalate Fabrics with Focus on Tensile Strength Degradation

Hastia Asadi [1,*,†], Joerg Uhlemann [1,†], Natalie Stranghoener [1,†] and Mathias Ulbricht [2,†]

[1] Institute for Metal and Lightweight Structures, University of Duisburg-Essen, Universitaetsstr. 15, 45141 Essen, Germany; joerg.uhlemann@uni-due.de (J.U.); natalie.stranghoener@uni-due.de (N.S.)
[2] Lehrstuhl für Technische Chemie II, University of Duisburg-Essen, Universitaetsstr. 7, 45117 Essen, Germany; mathias.ulbricht@uni-essen.de
* Correspondence: hastia.asadi@uni-due.de
† These authors contributed equally to this work

Table S1. Absorption peaks of polyethylene-terephthalate (PET).

Absorption Peak (cm^{-1})	Characteristic
3540	Stretching vibration of O-H in hydroxyl end group (hydrolysis product)
3480	O-H of carboxylic acid
3290	Stretching vibration of O-H of the carboxyl end (hydrolysis product)
3256	Carboxyl end group
2953	Asymmetric stretching vibration of C-H of methylene (-CH_2- in ethylene glycol) and stretching aliphatic vibration in the amorphous region
2864	Symmetric stretching vibration of C-H of methylene (-CH_2- in ethylene glycol) and stretching aliphatic vibration in the crystalline region
2650	-C-H of aldehyde
2258	Extent of crystallization (hydrolysis)
1760	C=O of aldehyde
1720	C=O of ester
1711	C=O of carboxylic acid, and carbonyl stretching as a signature of chain scission
1688	C=O of COOH
1576	Stretching vibration of C=O carbonyl group of carboxyl acid
1454	Bending (scissoring) vibration of C-H of the methylene (-CH_2- in ethylene glicol), bending of –CH_2- in the ethylene glycol in amorphous area, gauche conformer of ethylene glycol
1376	Wagging vibration of C-H of the methylene (-CH_2- in ethylene glycol), wagging vibration of –CH_2- in the ethylene glycol segment in the amorphous region, gauche conformer of ethylene glycol
1343	Wagging vibration of C-H of the methylene (-CH_2- in ethylene glycol), wagging vibration of –CH_2- in the ethylene glycol segment in the crystalline region, trans conformer of ethylene glycol
1233	C-O of carboxylic acid (by product of photodegradation in both Norrish type I and II), stretching vibration of C-O of carboxylic acid
1090	Symmetric stretching vibration of C-O of ethylene glycol (O-CH_2), and gauche form in the amorphous region
1018	In plane ring deformation (C–H) in the amorphous phase
972	Asymmetric stretching vibration of C-O of ethylene glycol (O-CH_2), and trans form in the crystalline region
898	Rocking vibration of C-H of the methylene (-CH_2-) in the ethylene glycol) in the amorphous region, gauche form of ethylene glycol
837	Rocking vibration of C-H of the methylene (-CH_2-) in the ethylene glycol) in the crystalline region, trans form of ethylene glycol
718	Out of plane bending vibration of ring (C-H), in-plane bending vibration of benzene ring
498	Stretching vibration of C-C between ring and ester group
1343/1376	Crystallinity index
100 × (973/1018)	Degree of crystallinity

Table S2. Direct and multi-step pathway equations for the tensile strength deterioration of PET type II under artificial exposure M4, t: time of exposure (hour) and Ts: tensile strength.

**Warp Direction**	
$t \rightarrow Ts$	$Ts = -6 \times 10^{-8} t^3 + 1.7 \times 10^{-4} t^2 - 0.17t + 62.57$
$t \rightarrow M_n$	$M_n = -6.26 \times 10^{-6} t^3 + 0.024t^2 - 19.13t + 26802.46$
$M_n \rightarrow Ts$	$Ts = 0.01\, M_n - 142.05$
$t \rightarrow M_n \rightarrow Ts$	$Ts = -4.72 \times 10^{-8} t^3 + 1.42 \times 10^{-4} t^2 - 0.14t + 60.04$
$t \rightarrow$ Chain scission	$Cs = 1.9 \times 10^{-10} t^3 - 6.3 \times 10^{-7} t^2 + 8.1 \times 10^{-4} t + 0.06$
Chain scission $\rightarrow Ts$	$Ts = -137.57Cs + 65.24$
$t \rightarrow$ Chain scission $\rightarrow Ts$	$Ts = -2.66 \times 10^{-8} t^3 + 8.7 \times 10^{-5} t^2 - 0.11t + 57.38$
$t \rightarrow C=O$ of COOH	$CO = -5.4 \times 10^{-12} t^3 - 2.46 \times 10^{-8} t^2 + 7.5 \times 10^{-5} t + 1.48$
$C=O$ of COOH $\rightarrow Ts$	$Ts = -2355.57CO^2 + 6132.08CO - 3863.9$
$t \rightarrow C=O$ of COOH $\rightarrow Ts$	$Ts = 4.87 \times 10^{-13} t^4 + 1.3 \times 10^{-8} t^3 + 7.6 \times 10^{-6} t^2 - 0.06t + 49.64$
**Weft direction**	
$t \rightarrow Ts$	$Ts = 2 \times 10^{-9} t^3 + 2 \times 10^{-5} t^2 - 0.05t + 31.873$
$t \rightarrow M_n$	$M_n = -2.6 \times 10^{-6} t^3 + 0.01t^2 - 14.82t + 25217.39$
$M_n \rightarrow Ts$	$Ts = 0.003M_n - 55.12$
$t \rightarrow M_n \rightarrow Ts$	$Ts = -8.86 \times 10^{-9} t^3 + 3.28 \times 10^{-5} t^2 - 0.050t + 30.64$
$t \rightarrow$ Chain scission	$Cs = -1.11 \times 10^{-10} t^3 + 8.5 \times 10^{-8} t^2 + 5.13 \times 10^{-4} t + 0.09$
Chain scission $\rightarrow Ts$	$Ts = -52.49Cs + 33.1$
$t \rightarrow$ Chain scission $\rightarrow Ts$	$Ts = 5.85 \times 10^{-9} t^3 - 4.47 \times 10^{-6} t^2 - 0.03t + 28.46$
$t \rightarrow C=O$ of COOH	$CO = -7.23 \times 10^{-12} t^3 + 2.18 \times 10^{-8} t^2 - 2.16 \times 10^{-6} t + 0.52$
$C=O$ of COOH $\rightarrow Ts$	$Ts = -5790.49CO^2 + 5089.8CO - 1059.81$
$t \rightarrow C=O$ of COOH $\rightarrow Ts$	$Ts = -2.9 \times 10^{-12} t^4 + 7.26 \times 10^{-9} t^3 - 2.03 \times 10^{-5} t^2 + 0.002t + 21.42$
$t \rightarrow$ Crystallinity	$cry = -1.4 \times 10^{-8} t^2 - 6.9 \times 10^{-6} t + 1$
Crystallinity $\rightarrow Ts$	$Ts = 17426.75cry^2 - 33555.01cry + 16152.3$
$t \rightarrow$ Crystallinity $\rightarrow Ts$	$Ts = 3.35 \times 10^{-12} t^4 + 4.18 \times 10^{-9} t^3 - 1.77 \times 10^{-5} t^2 - 0.01t + 24.38$
